# 12-week preoperative probiotic supplementation versus placebo: effects on inflammation, endotoxemia, adipokines, and gastrointestinal peptides in patients six months after bariatric surgery – a double-blind, randomized, placebo-controlled clinical trial

**DOI:** 10.1186/s12937-025-01217-2

**Published:** 2025-10-09

**Authors:** Marta Potrykus, Marcin Stanisławowski, Sylwia Czaja-Stolc, Anna Potrykus, Marta Stankiewicz, Anna Owczarzak, Marek Guzek, Michał Szymański, Igor Łoniewski, Krystian Adrych, Sylwia Małgorzewicz, Łukasz Kaska, Tomasz Ślebioda, Monika Proczko-Stepaniak

**Affiliations:** 1 Department of Oncological, Transplant, and General Surgery, Medical University of Gdansk, Gdansk, Poland; 2 Department of Histology, Medical University of Gdansk, Gdansk, Poland; 3 Department of Clinical Nutrition and Dietetics, Medical University of Gdansk, Gdansk, Poland; 4 Faculty of Philology, Institute of English and American Studies, University of Gdansk, Gdansk, Poland; 5 Department of Gastroenterology and Hepatology, Medical University of Gdansk, Gdansk, Poland; 6 Sanprobi Sp. Z O.O. Sp. K., 70-535 Szczecin, Poland; 7 Department of Biochemical Science, Pomeranian Medical University in Szczecin, Szczecin, Poland; 8 Independent Public Health Care Center of the Ministry of Internal Affairs and Administration, Gdansk, Poland

**Keywords:** Obesity, Bariatric surgery, Microbiota, Probiotics, Adipokines, Inflammation

## Abstract

**Background:**

Disruption in gut microbiota has been identified as a contributor to obesity-related inflammation and metabolic disorders. This study investigates the effects of preoperative probiotic supplementation on inflammation, endotoxemia, adipokines, and gastrointestinal peptides after bariatric surgery.

**Methods:**

This randomized, double-blind, placebo-controlled clinical trial included patients undergoing laparoscopic sleeve gastrectomy (LSG) or one anastomosis gastric bypass (OAGB). Participants were randomized to receive a 12-week supplementation of either a probiotic mixture, Sanprobi Barrier, which contained nine strains of bacteria (*Bifidobacterium bifidum* W23, *Bifidobacterium lactis* W51 and W52, *Lactobacillus acidophilus* W37, *Levilactobacillus brevis* W63, *Lacticaseibacillus casei* W56, *Ligilactobacillus salivarius* W24, *Lactococcus lactis* W19, and *Lactococcus lactis* W58), or a placebo before surgery. The key outcomes measured at baseline and 6 months postoperatively included serum lipopolysaccharide (LPS), cytokines (interleukin-6 – IL-6, interleukin-2 receptor—IL-2R, and C-reactive—CRP protein), adipokines (leptin, adiponectin, resistin), and gastrointestinal peptides (glucagon-like peptide-1 – GLP-1, ghrelin, and trefoil factor 2). Relative mRNA expression of ghrelin and trefoil family factor 2 in gastric tissues was also analyzed at baseline and on the day of the surgery.

**Results:**

Out of the initial 110 participants, serum samples of 18 individuals in the probiotic group and 24 in the placebo group were analyzed. Both groups showed significant reductions in serum LPS levels six months after surgery; however, no significant differences were observed between the two groups. Adiponectin levels increased significantly in the placebo group (4.2 ± 2.3 vs. 2.2 ± 1.1 pg/mL; *p < *0.001), while leptin levels decreased significantly in both groups without intergroup differences. IL-6 levels were significantly lower in the probiotic group compared to the placebo group at 6 months (2.2 ± 1.1 vs 4.2 ± 2.3 pg/mL; *p* = 0.004). No significant differences were observed in the remaining cytokine levels between the groups. Gastrointestinal peptides showed no significant differences between the groups, although GLP-1 levels improved within both groups. No changes were observed in ghrelin and trefoil factor 2 expression at the mRNA level.

**Conclusions:**

Preoperative probiotic therapy was associated with significantly lower IL-6 levels compared to placebo six months after surgery, suggesting a potential anti-inflammatory effect. However, since the between-group difference in IL-6 changes from baseline was not statistically significant, the observed effect should be interpreted with caution. Other measured markers were not significantly affected, though low statistical power may have limited detection of subtle effects. These findings suggest that while probiotics may reduce certain inflammatory responses, their efficacy can be overshadowed by bariatric surgery impact. The further studies on this subject are warranted.

**Trial registration:**

The study was registered at ClinicalTrials.gov (NCT05407090).

## Background

Obesity is a complex global health problem that demands comprehensive research across all dimensions, including its molecular basis [1]. The complex interaction between gastrointestinal peptides, cytokines, adipokines, and the intestinal microbiota offers a promising area for discovering the fundamental mechanisms that regulate metabolic processes. In recent years, research has increasingly highlighted the role of microbiota and its metabolites in various aspects of human physiology, comprising energy metabolism, metabolic regulation, and immune function [2].

An imbalance in the intestinal microbiota may increase intestinal permeability and allow excessive passage of structural parts of microorganisms, including lipopolysaccharide (LPS), into the bloodstream. LPS binding to Toll-like receptors (TLRs) on the surface of enteroendocrine cells (ECCs), immune cells, and adipocytes triggers the synthesis of pro-inflammatory cytokines and adipokines. Low-grade inflammation causes the intensification of intestinal barrier disorders, further increasing the translocation of harmful substances from the intestinal lumen. Endotoxins, by activating TLR4 receptors on the surface of adipocytes, can alter their secretion and intensify the inflammation of adipose tissue associated with obesity [3, 4]. The disrupted adipokine profile can contribute to metabolic disturbances [5].

Additionally, the microbiota interacts with intestinal epithelial cells, influencing their activities. Microbiota and bacterial-driven short-chain fatty acids (SCFAs) play a crucial role in this interaction by influencing hormones such as glucagon-like peptide-1 (GLP-1), ghrelin, and trefoil factor family 2 (TFF2) [6, 7]. These hormones improve glucose metabolism and suppress appetite, contributing to the maintenance of optimal body weight [5]. Additionally, the TFF2 protein stabilizes the mucus barrier and regulates the repair processes of the gastrointestinal epithelium [8]. Disruptions in these mechanisms may lead to obesity and metabolic disorders.

In our previous work [9], preoperative probiotic supplementation did not affect the clinical outcomes of surgical treatment of obesity. Nevertheless, it is possible that the effect of the intervention was too subtle to manifest in detectable clinical effects. However, it may have contributed to underlying mechanisms involved in the pathogenesis and management of obesity and metabolic disorders. Therefore, the present study aimed to determine whether preoperative probiotic supplementation influences metabolic processes at the molecular level following bariatric surgery (BS), focusing on endotoxemia, inflammation, adipokines, and gastrointestinal peptides. Investigating the impact of gut microbiota modulation on these compounds may clarify new pathways and mechanisms underlying obesity and metabolic disorders. Understanding these interactions at the molecular level may advance therapeutic strategies aimed at targeting obesity and related metabolic disorders. Therefore, the study was designed to assess whether a 12-week preoperative probiotic therapy would impact the long-term (six month) postoperative levels of gut microbiota metabolite LPS, as well as compounds involved in energy metabolism regulation and the immune system — ghrelin, GLP-1, interleukin-6 (IL-6), interleukin-2 receptor (IL-2R), C-reactive protein (CRP), adiponectin, leptin, and resistin — along with the less explored protein TFF2, which plays a role in regulating gastric epithelial barrier. Another objective of the study was to examine if probiotic administration influences mRNA expression of the genes encoding ghrelin and TFF2.

## Methods

### Study design

The study was structured as a randomized, double-blind, placebo-controlled clinical trial with a 12-week period of probiotics intervention. Participants were randomly assigned into two research arms – the probiotic and the placebo group. Allocation to these groups was carried out in a 1:1 ratio using a Microsoft Excel version 2019 random number generator. The study was unblinded after the completion of the statistical analysis. Randomization, blinding, and unblinding of the study were performed by an independent researcher not involved in conducting the study. The project was executed between August 2021 and April 2023 at the University Clinical Center (UCC) in Gdańsk, Poland. The protocol obtained approval from the Independent Bioethics Committee for Scientific Research at the Medical University of Gdańsk (Approval No. NKNNB/447/2021) on May 21, 2021, in accordance with the Declaration of Helsinki. The study was registered with Clinical Trials with the registration number NCT05407090.

### Participants

As described in our previous research [9], one hundred ten patients qualified for BS were enrolled in our study after screening interviews. The patients were qualified for Laparoscopic Sleeve Gastrectomy (LSG) or One Anastomosis Gastric Bypass (OAGB). The decision to perform either OAGB or LSG was determined through a shared decision-making process between physician and patient, reflecting an individualized approach based on the patient’s overall health, with particular emphasis on metabolic comorbidities, baseline body weight, and the presence of gastroesophageal reflux disease. The inclusion criteria included eligibility for BS based on International Federation for the Study of Obesity (IFSO) guidelines [10], Caucasian race, and age over 18. Patients were excluded from the study if they met any of the exclusion criteria: allergy/intolerance to any of the ingredients of the preparations; inflammatory bowel diseases; current antibiotic therapy; immunosuppression; biological treatment; long-term antibiotic therapy; probiotic therapy in the 1 month before study enrollment; neurodegenerative diseases, and antipsychotic drugs. Surgical treatments were performed in accordance with the Enhanced Recovery After Bariatric Surgery (ERABS) protocol [11].

### Sample size/power calculations

This study is part of a broader clinical trial, with the primary outcome being body weight loss six months after bariatric surgery. The sample size calculation was therefore based on anticipated differences in weight loss, which were reported in our primary publication [9]. This is the first study to assess the effects of preoperative probiotic supplementation in bariatric patients; therefore, no prior data were available to guide a direct sample size calculation. We based our estimate on weight reduction outcomes reported by Sánchez et al. [12] and calculated in study protocol [13]. Assuming α = 0.05 and 80% power, the initial calculation indicated that 20 patients per group would be sufficient. To enhance study reliability and account for protocol deviations and dropouts, we increased the planned sample size to 55 participants per group (total n = 110).

The current analysis focuses on secondary outcomes, including markers of inflammation, endotoxemia, adipokines, and gastrointestinal peptides. Since these were not used in the original sample size calculation, a post-hoc power analysis was conducted using the final sample sizes (*n* = 24 for the placebo group and *n* = 18 for the probiotic group). To account for multiple comparisons, a Bonferroni-corrected significance level of α = 0.005 was applied. The power was calculated using the TTestIndPower function from the statsmodels library in Python. The calculated power for each variable was as follows: Leptin: 3.3%, Adiponectin: 1.2%, Resistin: 3.7%, IL-2R: 1.0%, IL-6: 66.6%, CRP: 1.8%, LPS: 3.2%, GLP-1: 4.4%, TFF2: 0.5%, and Ghrelin: 3.7%.The power of the test for IL-6 was moderate (66.6%), indicating a reasonable chance of detecting a true effect. For the other variables, the power was low (< 50%), suggesting difficulty in detecting true effects with the stringent significance criteria applied. By adjusting our significance level and calculating the statistical power accordingly, we aimed to minimize the risks of both Type I and Type II errors, thereby enhancing the reliability of our findings.

### Study protocol

**Fig. 1:**
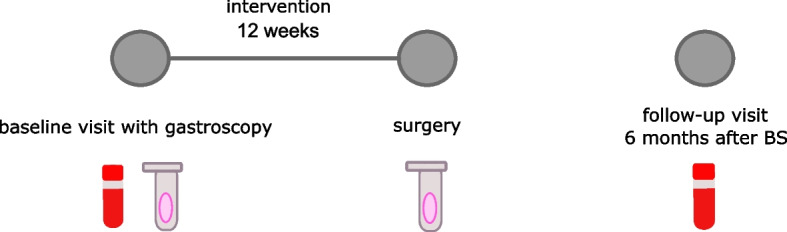
Study design overview

Baseline and 6-month post-surgery evaluations were conducted at the UCC in Gdańsk. Although the treatment plan includes 3 postoperative visits within 6 months after surgery, due to the limited number of serum samples collected from patients at other time points, we measured the assessed parameters only at the 6-month postoperative time point. Throughout the study, both groups received the same medical care. Anthropometric measurements and blood samples were obtained during both evaluations. After 12 weeks of supplementation, the patients underwent either LSG or OAGB surgery. Gastroscopy, during which tissue samples were collected, was performed before the intervention. Patients who tested positive for *Helicobacter pylori* were instructed to refrain from starting supplementation until the completion of the bacterial eradication therapy. Additional tissue samples were collected during surgery, which was systematically scheduled approximately three months post-intervention to allow for a 12-week treatment period. Since bariatric treatment must be adapted to the patient’s condition and needs, the treatment timeline could be modified, and the surgery postponed to provide optimal medical care. For this reason, only patients with at least 4 weeks of supplementation were included in the analysis. Patients adhered to a standardized diet under the guidance of a qualified dietitian. More detailed information on the patients' diet is presented in our previous publication [9]. A simplified study design is presented in Fig. 1.

### Intervention

The study product was a probiotic mixture containing nine bacterial strains: *Bifidobacterium bifidum* W23, *Bifidobacterium lactis* W51 and W52, *Lactobacillus acidophilus* W37, *Levilactobacillus brevis* W63, *Lacticaseibacillus casei* W56, *Ligilactobacillus salivarius* W24, *Lactococcus lactis* W19, and *Lactococcus lactis* W58, with a daily dose of 2 × 10⁹ colony forming units (CFU). This product is marketed in Poland as Sanprobi Barrier (Sanprobi sp. z o.o. sp. k., Szczecin, Poland) and is approved by relevant health authorities for its composition and recommended use. The placebo capsules, identical in appearance, contained maize starch and maize-derived maltodextrin. Patients were instructed to store both products in the refrigerator and take four capsules daily with meals (two in the morning and two in the evening) for 12 weeks. Supplementation was to continue until the last meal before surgery, within 24 h before the procedure.

### Venous blood collection

Fasting venous blood samples were collected at the Central Laboratory of the UCC. To prepare serum samples, blood was drawn directly into a centrifuge tube without any anticoagulant, left at room temperature for 30 min to allow clotting, and then centrifuged at 3000xg for 15 min at 4 °C. The resulting serum samples were aliquoted and stored at − 80 °C for subsequent analyses.

### Tissue collection

Before the intervention, during routine gastroscopy, stomach’s body samples approximately 2mmx2mm in size were collected. The tissues were placed in a sterile empty tube and frozen at –80^○^C until analysis. After the intervention, tissues were collected during surgery and stored under the same conditions. In both cases, the patients were fasting and standardly prepared for the above-mentioned procedures. Tissues were collected to determine gene expression of ghrelin and TFF2 at the mRNA level.

### Adipokines, cytokines, and gastrointestinal peptides in serum

Laboratory tests were performed to determine the levels of adiponectin, leptin, resistin, LPS, GLP-1, acylated ghrelin, and TFF2 using an Enzyme-Linked Immunosorbent Assay (ELISA kit). Inflammatory markers, such as IL-6, IL-2R, and CRP, were assayed at the certificated laboratory of the UCC in Gdańsk.

### mRNA expression

#### Sample collection and processing

A segment of the stomach was aseptically excised and immediately frozen at − 80 °C for subsequent gene expression analyses. The tissue was then homogenized in fenozol buffer using the Total RNA Mini Kit (A&A Biotechnology, Gdynia, Poland) in the presence of zirconium beads (Benchmark Zirconium Beads, Merck). The homogenization process was performed with the MagNA Lyser device (Roche, Basel, Switzerland) at 7000 vibrations per minute for 90 s.

#### RNA extraction and quantification

Total RNA was extracted from the samples with the Total RNA Mini kit (A&A Biotechnology, Gdynia, Poland). The RNA concentration was determined using the NanoDrop 2000 spectrophotometer (Thermo Scientific, USA), which verified purity by assessing the absorbance ratio at 260 to 280 nm wavelengths. Only samples with an absorbance ratio between 1.9 and 2.1 were included in subsequent analyses. The RNA concentration was adjusted to 1 μg/μL before reverse transcription, which was performed using the PrimeScript RT Reagent Kit (Takara, Dalian, China) according to the manufacturer’s protocol.

#### Real-time quantitative PCR

Gene expression analysis for the internal standard (Glyceraldehyde-3-phosphate dehydrogenase — GAPDH), ghrelin, and TFF2 was performed employing qRT-PCR from synthesized cDNA. PCR reactions were prepared in 20 μL volumes containing 10 μL of TaqMan Universal PCR Master Mix (Thermo Fisher Scientific, Waltham, MA, USA), 1 μL cDNA, and TaqMan probes for each target gene (Ghrelin – Hs01074053_m1, TFF2 — Hs00193719_m1, GAPDH — Hs99999905_m1) obtained from Thermo Fisher following the manufacturer’s protocol. The qRT-PCR was conducted utilizing a Step One thermocycler (Applied Biosystem, USA). The temperature–time profiles included an initial denaturation at 95 °C for 30 s, followed by 40 cycles at 95 °C for 5 s, 60 °C for 20 s, and 72 °C for 30 s, with fluorescence detection during the extension step of each cycle. All reactions were performed in duplicate.

#### Data analysis

Analysis of qRT-PCR data was executed using the Livak and Schmittgen 2 − ΔΔCt method [13]. The normalized gene expression at the mRNA level of each target gene, normalized to the ΔCt of the housekeeping gene (GAPDH), was calculated using the equation: normalized gene expression = 2^-(Ct target gene – Ct housekeeping gene).

### Statistical analysis

Various statistical methods were employed depending on the data type and distribution Means and SDs were used for data with a normal distribution, while medians and interquartile ranges (IQRs) were utilized for non-normally distributed data. The Shapiro–Wilk test and histogram visualizations were used to check for normality. Categorical data were compared using contingency tables and either chi-squared tests or Fisher’s exact test (for 2 × 2 tables), for small sample sizes (< 5). Normally distributed continuous data were compared using t-tests or Welch's correction if variances were unequal (Brown-Forsythe test < 0.05). For non-normally distributed continuous data, Mann–Whitney U tests were applied. Paired samples t-tests were used to assess within-group parameter changes, with the Wilcoxon signed-rank test utilized when normality assumptions were violated. To control the risk of false positives due to multiple comparisons, we employed an adjusted significance level using the Bonferroni correction. The standard significance level of α = 0.05 was divided by the number of tests conducted, resulting in an adjusted significance level of less than 0.005 for all two-sided tests. Statistical analyses and graphical representations were conducted using GraphPad Prism 10.2.1, JASP statistical software version 0.18.1, and Python version 3.9.16.

## Results

**Fig. 2:**
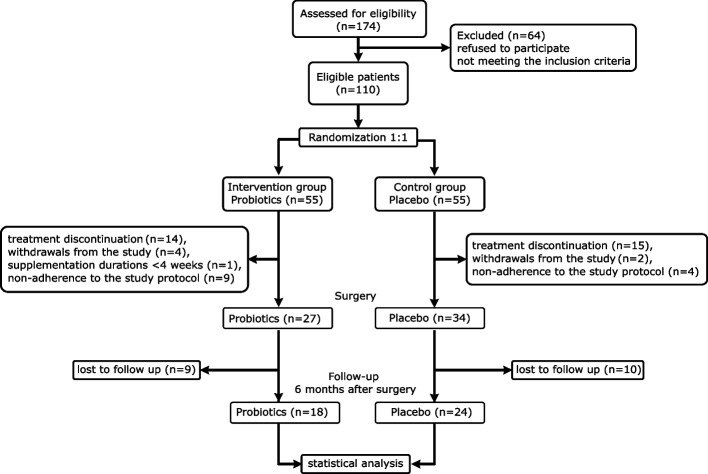
Flow chart

The study initially enrolled 110 participants; however, for the statistical analysis of serum parameters, 18 patients from the probiotic group and 24 from the placebo group were included (Fig. 2).

All baseline characteristics and clinical outcomes were comparable between the two groups, as indicated in Tables 1 and 2. None of the evaluated parameters demonstrated significant differences between the groups before the intervention (Table 3).

**Table 1:** Characteristic of groups before intervention (serum)

	Placebo (*n* = 24)	Probiotics (*n* = 18)	*p*-value
Sex (F/M) n	18/6	12/6	0.554
Age [years]	39.6 ± 10.7	38.7 ± 10.9	0.787
Duration of supplementation [weeks]	10.7 ± 2.1	9.8 ± 2.3	0.200
Max weight [kg]	139.8 ± 23.5	132.1 ± 21.3	0.283
Max BMI [kg/m^2^]	46.9 ± 5	44.7 ± 6	0.206
Type of surgery (LSG/OAGB)	22/2	15/3	0.636^#^
Current-smokers n (%)	8 (33.3)	0 (0)	0.006
Ever-smokers n (%)	9 (37.5)	6 (33.3)	0.780
DM1 n (%)	0 (0)	0 (0)	1
DM2 n (%)	5 (20.8)	5 (27.8)	0.601
HTN n (%)	11 (45.8)	9 (50)	0.789
DL n (%)	11 (45.8)	5 (27.8)	0.233
HT n (%)	9 (37.5)	6 (33.3)	0.78
Fatty liver n (%)	20 (83.3)	13 (72.2)	0.462^#^
OSAS (%)	16 (66.7)	12 (66.7)	1
Impaired fasting glucose n (%)	6 (25)	7 (38.9)	0.335

*Abbreviations*: *BMI* Body Mass Index, *LSG* Laparoscopic Sleeve Gastrectomy, *Female* Female, *M* Male, *OAGB* One Anastomosis Gastric Bypass, *DM1* Diabetes Mellitus Type 1, *DM2* Diabetes Mellitus Type 2, *HTN* Hypertension, *DL* Dyslipidemia, *HT* Hypothyroidism, *OSAS* Obstructive Sleep Apnea Syndrome,

^#^Fisher's exact test

**Table 2:** Clinical outcomes before intervention and 6 month after surgery

Baseline				6 M PostOP			Change within group *p*-value		
	Placebo (*n* = 24)	Probiotics (*n* = 18)	*p*-value	Placebo (*n* = 24)	Probiotics (*n* = 18)	*p*-value	Placebo (*n* = 24)	Probiotics (*n* = 18)	Both groups (*n* = 42)
Weight [kg]	132.2 ± 22.8	122.7 ± 25.6	0.212	95.3 ± 17.3	88.2 ± 20.1	0.228	< 0.001	< 0.001	< 0.001
BMI [kg/m^2^]	44.3 ± 4.6	41.3 ± 6.8	0.101	32 ± 4.7	29.7 ± 5.5	0.152	< 0.001	< 0.001	< 0.001
Vit. D [pg/ml]	49.6 ± 15.3	51.4 ± 17	0.716	61.6 ± 20.4	57.9 ± 15.9	0.525	0.003	0.218	0.002
Folic Acid [ng/ml]	5.9 ± 1.9	7.3 ± 2.6	0.045	12.1 ± 8.6	8.8 ± 3.6	0.129	0.002	0.201	< 0.001
Vit. B12 [pg/ml]	331.5 ± 105.1	355.9 ± 131.4	0.507	407.4 ± 143.7	377.7 ± 158.7	0.529	0.026	0.521	0.029
Iron [ug/dl]	72.5 ± 31.6	70.8 ± 31.2	0.865	85.5 ± 20.4	101.5 ± 34.5	0.068	0.037	0.009	< 0.001
Insulin [uU/ml]	20.6 ± 15.6	19.6 ± 18.6	0.838	7.5 ± 2.2	7.9 ± 6.2	0.795	< 0.001	0.01	< 0.001
LDH [U/l]	192.8 ± 44	192.6 ± 36.1	0.988	158.9 ± 34.4	164.1 ± 38.1	0.648	< 0.001	0.004	< 0.001
ALT [U/l]	36 ± 18.2	30.6 ± 15.2	0.314	17.6 ± 6.9	19.3 ± 8.7	0.5	< 0.001	0.002	< 0.001
AST [U/l]	23.5 ± 9.4	21.7 ± 9	0.55	16.7 ± 5	17.7 ± 7.1	0.591	< 0.001	0.122	< 0.001
GGT [U/l]	41.4 ± 26.2	33.2 ± 18	0.258	20.8 ± 16.9	35.8 ± 60.2	0.249	< 0.001	0.831	0.075
ALP [U/l]	84.8 ± 25.7	72.9 ± 12.8	0.082	79.8 ± 24.8	74.6 ± 22.4	0.493	0.294	0.695	0.509
TG [mg/dl]	156.3 ± 77.8	126.4 ± 60.2	0.184	109.3 ± 44.3	98.3 ± 43	0.427	< 0.001	0.068	< 0.001
HDL [mg/dl]	43.3 ± 8.7	46.6 ± 13	0.335	46.3 ± 7.8	52.9 ± 10.7	0.024	0.121	0.01	0.003
LDL [mg/dl]	114 ± 41.4	113.5 ± 20.7	0.966	116.6 ± 36	117.1 ± 33.9	0.96	0.758	0.592	0.583
Cholesterol [mg/dl]	185.3 ± 47.9	183.2 ± 32	0.877	171.4 ± 46.5	183.8 ± 41	0.376	0.165	0.947	0.25
HbA1c%	5.7 ± 0.6	5.9 ± 1.3	0.375	5.3 ± 0.5	5.2 ± 0.3	0.64	< 0.001	0.031	< 0.001
HbA1c [mmol/mol]	38.3 ± 6.9	41.3 ± 14	0.352	34.1 ± 5.1	33.4 ± 3.7	0.582	< 0.001	0.033	< 0.001
Glucose [mg/dl]	101 ± 17.9	108.9 ± 43.2	0.419	89.2 ± 11.6	87.9 ± 12.6	0.747	< 0.001	0.046	0.001
Hemoglobin [g/dl]	13.8 ± 1.6	13.7 ± 1.8	0.901	13.8 ± 1.5	14.1 ± 1.2	0.493	0.987	0.35	0.469
HOMA-IR	5.5 ± 5.8	5.7 ± 6.7	0.94	1.6 ± 0.7	1.8 ± 2	0.64	0.003	0.011	< 0.001

*Abbreviations: BMI* Body Mass Index, *Vit. D* Vitamin D, *Vit. B*_*12*_ Vitamin B_12_, *LDH* Lactate dehydrogenase, *ALT* Alanine aminotransferase, *AST* Aspartate aminotransferase, *GGT* Gamma-glutamyl transferase, *ALP* Alkaline phosphatase, *TG* triglycerides, *HDL* High-density lipoprotein, *LDL* Low-density lipoprotein, *HbA1c%* glycated hemoglobin percentage, HbA1c—glycated hemoglobin, *HOMA-IR* Homeostatic model assessment for insulin resistance

**Table 3:** Serum adipokines, inflammatory factors, and peptide concentrations of patients in both groups over the study period

	Placebo (*n* = 24)	Probioticsm (*n* = 18)	*p*-value^a^
Leptin [ng/mL]			
Baseline	41.4 ± 15.8	35.9 ± 15.3	0.260
6 M PostOP	19.2 ± 14.8	14.9 ± 12.3	0.333
MD (95% CI), P^*^	−22.3 (−29.1, −15.4) < 0.001	−20.9 (−26.2, −15.6) < 0.001	0.760
Adiponectin [ng/mL]			
Baseline	6354.1 ± 2113.7	8290.6 ± 2937.1	0.017
6 M PostOP	8921.2 ± 3022	9397.7 ± 2248.1	0.585
MD (95% CI), P^*^	2567.2 (1758.5, 3375.8) < 0.001	1265.7 (364.7, 2166.7) 0.009	0.032
Resistin [ng/mL]			
Baseline	8 ± 3.9	6.6 ± 2.1	0.137^#^
6 M PostOP	7.7 ± 3.3	6.7 ± 3	0.287
MD (95% CI), P^*^	−0.2 (−2.1, 1.6) 0.781	0.1 (−1.3, 1.5) 0.885	0.769
IL-2R [U/mL]			
Baseline	404.5 ± 113.8	429.1 ± 159.5	0.562
6 M PostOP	403.8 ± 130.2	385.1 ± 123.5	0.641
MD (95% CI), P^*^	−0.7 (−37.4, 36.1) 0.970	−44 (−105.6, 17.7) 0.151	0.191
IL-6 [pg/mL]			
Baseline	3.2 ± 1.4	3.2 ± 1.4	0.994
6 M PostOP	4.2 ± 2.3	2.2 ± 1.1	0.002
MD (95% CI), P^*^	1 (−0.04, 2.1) 0.058	−1 (−1.9, 0) 0.049	0.008
CRP [mg/L]			
Baseline	8.4 ± 5.6	6.8 ± 5.3	0.348
6 M PostOP	2.8 ± 3.4	2.1 ± 2.3	0.449
MD (95% CI), P^*^	−5.6 (−7.6, −3.6) < 0.001	−4.7 (−7.1, −2.3) < 0.001	0.545
LPS [pg/mL]			
Baseline	279 ± 91.7	285.3 ± 87	0.825
6 M PostOP	63 ± 53.3	47.6 ± 45	0.330
MD (95% CI), P^*^	−216.1 (−255.6, −176.5) < 0.001	−237.6 (−274.2, −201) < 0.001	0.424
GLP-1 [pg/mL]			
Baseline	3.6 ± 3.4	3.4 ± 1.9	0.853
6 M PostOP	19 ± 21.1	12.3 ± 5.7	0.2
MD (95% CI), P^*^	15.4 (7.3, 23.4) < 0.001	8.9 (5.8, 12) < 0.001	0.173
TFF2 [ng/mL]			
Baseline	309.1 ± 168.6	219.1 ± 164.7	0.094
6 M PostOP	253.1 ± 429.7	271.1 ± 389.2	0.891
MD (95% CI), P^*^	−53.6 (−243.6, 136,4) 0.564	52 (−163.2, 267.2) 0.617	0.445
Ghrelin [pg/mL]			
Baseline	157.4 ± 136.9	130.5 ± 66.4	0.448
6 M PostOP	631.9 ± 702.7	419.8 ± 426.8	0.278
MD (95% CI), P^*^	483.5 (188.5, 778.6) 0.003	288.6 (71.6, 505.6) 0.012	0.328

^*^based on paired samples t-test

^#^based on Welch's test

^a^based on Independent samples t-test

### Lipopolysaccharide

Referring to the findings in Table 3, both groups showed significant reductions in the LPS concentrations (*p < *0.001 for both). However, the differences in concentrations at 6 months after BS (63 ± 53.3 vs. 47.6 ± 45 pg/mL *p* = 0.226) or mean differences (−216.1 vs. −237.6 pg/mL; *p* = 0.066) between the groups were not statistically significant.

### Adipokines

Leptin levels decreased in both groups, but no significant differences were observed between groups after surgery. The increase in adiponectin levels after BS was significant in the placebo group (*p < *0.001). No differences were observed either within or between groups for resistin, as reflected in Table 3.

### Cytokines

Levels of IL-6 differed significantly between the groups at 6 months, with the probiotic group showing significantly lower levels than the placebo group (4.2 ± 2.3 vs. 2.2 ± 1.1 pg/mL; *p* = 0.004). The difference between groups in change of IL-6 levels was not statistically significant (*p* = 0.006). No significant change was observed in IL-6 levels within groups. No changes in IL-2R and CRP concentrations were observed within groups or between-groups, as evidenced by the results in Table 3.

### Gastrointestinal peptides

As illustrated in Table 3, the mean differences in GLP-1 were statistically improved within each group. However, they were not statistically significant between the research arms. Ghrelin concentrations were significantly reduced 6 months after surgery compared to baseline in the placebo group, but not in the probiotic group. However, there were no differences between groups. No within-group or between-group differences in serum TFF2 concentration were observed.

### mRNA expression of genes

**Fig. 3:**
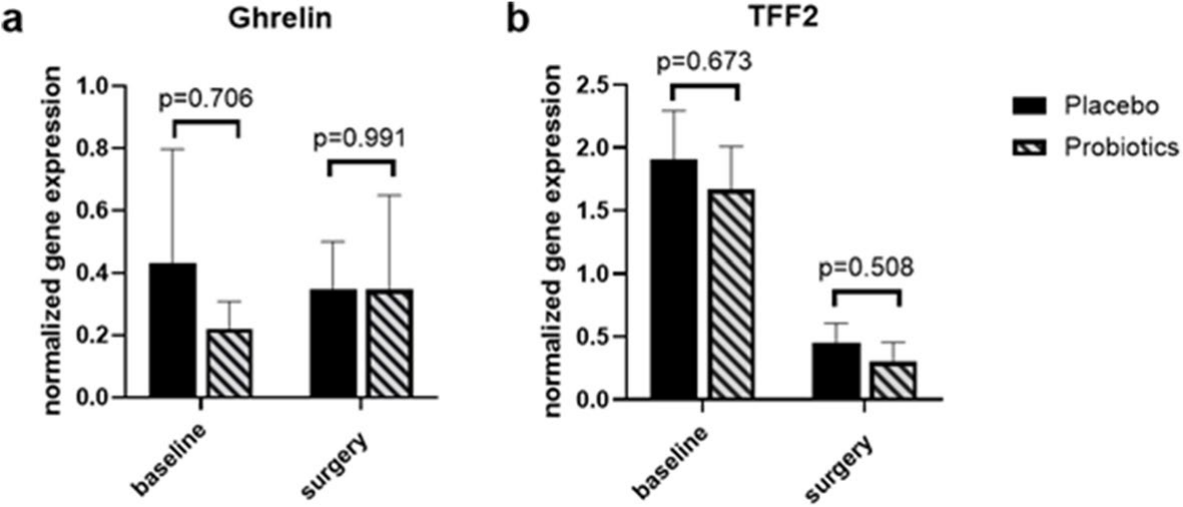
Comparison of the mRNA expression of ghrelin (**a**) and TFF2 (**b**) genes. Data presented as mean ± SEM

The analysis was conducted on gene expression at the mRNA level in paired samples from patients, comparing tissues collected before (during gastroscopy) and after (during surgery) the intervention. For ghrelin, 25 paired samples were analyzed, including 17 from the placebo group and 8 from the probiotics group. For TFF2, 29 paired samples were assessed, with 17 from the placebo group and 12 from the probiotics group. The analysis of patient data revealed no significant differences in baseline characteristics between the groups, as shown in Table 4. Additionally, no significant differences in the expression levels of ghrelin or TFF2 were observed between the placebo and probiotics groups, either before or after the intervention. However, we observed a trend of decreased TFF2 expression after the preoperative preparation for bariatric surgery. In the placebo group, the decrease in TFF2 expression was statistically significant (*p* = 0.001), whereas in the probiotic group, despite the reduction, it did not reach statistical significance (*p* = 0.007). When considering all patients from both groups, a statistically significant decrease was still observed (*p < *0.001). No significant differences in ghrelin expression were observed within the groups (Fig. 3).

**Table 4:** Characteristics of the groups (stomach tissue)

	Ghrelin expression analysis			TFF2 expression analysis		
	Placebo (*n* = 17)	Probiotics (*n* = 8)	*p*-value	Placebo (*n* = 17)	Probiotics (*n* = 12)	*p*-value
Sex (F/M) n	13/4	4/4	0.359^#^	13/4	7/5	0.422^#^
Age [years]	43.6 ± 10.9	41.1 ± 14.3	0.671	43.3 ± 10.9	41.7 ± 12.1	0.698
Duration of supplementation [weeks]	11.1 ± 1.4	9.8 ± 2.1	0.077	11 ± 1.4	9.9 ± 2.5	0.106
Max weight [kg]	139.4 ± 20.9	131.2 ± 22.1	0.378	135.8 ± 20.9	135.4 ± 26.9	0.963
Baseline [kg]	133.4 ± 18.6	121.7 ± 26.2	0.211	129.2 ± 19.8	126.8 ± 30.2	0.801
Weight OP [kg]	120.8 ± 17.8	112.7 ± 24.5	0.357	117.1 ± 18	116.5 ± 25.2	0.941
Max BMI [kg/m^2^]	46.8 ± 4.8	42.7 ± 5	0.064	46.1 ± 4.7	44.4 ± 7	0.446
Baseline [kg/m^2^]	44.8 ± 4.2	39.3 ± 4.3	0.006	43.9 ± 4.5	41.4 ± 7.5	0.279
BMI OP [kg/m^2^]	40.6 ± 4.3	36.4 ± 4.1	0.031	39.8 ± 4.3	38.1 ± 5.6	0.349
Type of surgery (OAGB/LSG)	15/2	7/1	1^#^	15/2	10/2	1^#^
Current-smokers n (%)	3 (18)	0 (0)	0.527^#^	4 (24)	0 (0)	0.121^#^
Ever-smokers n (%)	4 (24)	3 (38)	0.640^#^	5 (29)	3 (25)	1^#^
DM1 n (%)	0 (0)	0 (0)	1^#^	0 (0)	0 (0)	1
DM2 n (%)	4 (24)	2 (25)	1^#^	4 (24)	2 (17)	1^#^
HTN n (%)	9 (53)	4 (50)	1^#^	10 (59)	6 (50)	0.638
DL n (%)	9 (53)	3 (38)	0.673^#^	9 (53)	4 (33)	0.451^#^
HT n (%)	5 (29)	2 (25)	1^#^	4 (24)	3 (25)	1^#^
Fatty liver n (%)	14 (82)	7 (88)	0.743	14 (82)	10 (83)	0.945
OSAS n (%)	10 (59)	6 (75)	0.432	10 (59)	8 (67)	0.668
Impaired fasting glucose n (%)	5 (29)	3 (38)	1^#^	5 (29)	6 (50)	0.260

*Abbreviations*: *BMI* Body Mass Index, *LSG* Laparoscopic Sleeve Gastrectomy, *Female* Female, *M* Male, *OAGB* One Anastomosis Gastric Bypass, *DM1* Diabetes Mellitus Type 1, *DM2* Diabetes Mellitus Type 2, *HTN* Hypertension, *DL* Dyslipidemia, *HT* Hypothyroidism, *OSAS* Obstructive Sleep Apnea Syndrome

^#^Fisher's exact test

## Discussion

To the best of our knowledge, this is the first study to investigate the influence of preoperative probiotic supplementation on compounds related to the mechanism of obesity in patients after BS. The objective of the presented research was to assess the effects of preoperative multistrain probiotic administration on endotoxemia, inflammation, adipokines, and gastrointestinal peptide levels 6 months after BS. Additionally, we studied the effect of probiotics at mRNA expression of the genes encoding ghrelin and TFF2.

The results of this study indicate that preoperative probiotic supplementation was associated with significantly lower IL-6 levels compared to placebo six months after bariatric surgery. Although the absolute IL-6 levels differed between groups at six months, the change from baseline was not significantly different, suggesting potential influence but not a definitive effect of probiotics. Additionally, we observed no differences between groups in the remaining parameters examined.

IL-6 is secreted by various cell types, including the liver, pancreas, central nervous system, skeletal muscle, and adipocytes, where it mediates distinct metabolic functions. As a pleiotropic cytokine, IL-6 has diverse effects on metabolism, depending on its site of secretion and the target cells involved [14, 15]. Obesity leads to the expansion and dysregulation of white adipose tissue (WAT), particularly visceral fat, resulting in altered adipokine secretion [16]. Notably, more than one-third of basal circulating IL-6 is released by white adipose tissue (WAT) [15]. It contributes to chronic low-grade inflammation, which disrupts insulin signaling pathways and exacerbates insulin resistance. This chronic inflammatory state is a key factor of metabolic syndrome, which includes conditions such as type 2 diabetes, dyslipidemia, and cardiovascular disease [17]. Adipocyte-derived IL-6 promotes the accumulation of adipose tissue macrophages (ATMs) and contributes to the altered secretion of adipokines and cytokines, leading to a disruption in homeostasis that favors insulin resistance and metabolic dysregulation [18]. Therefore, reducing chronically elevated IL-6 levels in patients with obesity could have multiple beneficial effects. It may improve insulin sensitivity by alleviating the inhibitory effects of SOCS-3 on insulin signaling pathways, thereby enhancing glucose uptake in muscle and liver, and reducing hyperglycemia and the risk of type 2 diabetes [19]. Additionally, reducing IL-6 could improve lipid profiles by promoting a more favorable balance of LDL and HDL cholesterol [20]. Consequently, reducing IL-6 levels could decrease the risk of cardiovascular diseases, which are the significant contributor to mortality among patients with obesity [21].

However, IL-6 can also exert beneficial effects in specific contexts. Its pro-inflammatory activity is crucial in initiating the immune response, protecting the host from various infections by promoting the activation and recruitment of immune cells to sites of infection or injury. This acute inflammatory response is a necessary defense mechanism that helps to eliminate pathogens and initiate tissue repair [22]. In subcutaneous adipose tissues, IL-6 can enhance leptin-mediated GLP-1 secretion, which supports glucose-stimulated insulin release [18]. During exercise, muscle-derived IL-6 suppresses ATM accumulation [23], enhances glucose metabolism, and stimulates the production of anti-inflammatory cytokines [24]. Additionally, exercise-induced IL-6 improves lipid metabolism by promoting fatty acid oxidation and increasing energy expenditure [25]. While the acute inflammatory actions of IL-6 are protective, chronic elevation of this cytokine can be detrimental to long-term metabolic health [19]. Therefore, reducing elevated levels of IL-6 may be beneficial in obesity; however, enhancing its positive, context-dependent effects could offer a more effective strategy for improving metabolic outcomes in bariatric patients.

Despite the reduction in IL-6 levels observed in this study, this does not necessarily translate into direct, measurable clinical benefits in the short term, as demonstrated in our previous work [9]. Clinical outcomes such as weight loss and improved metabolic parameters may require a longer period of time to show improvement. Moreover, metabolic processes and inflammatory responses are complex and can be modulated by many factors. Modulation of the gut microbiota may be one element influencing these processes, but their effects on clinical outcomes may be subtle or require synergy with other interventions. As the results of the present study show, bariatric surgery has a significant impact on the assessed parameters, overshadowing the effects of probiotic therapy.

The other studied compounds also play a crucial role in regulating metabolism and processes related to obesity. Obesity is characterized by dysregulation in hunger and satiety mechanisms. There is a complex feedback system between the gastrointestinal system and the brain during eating. This axis regulates both homeostatic and hedonic feeding behaviors, playing a crucial role in maintaining energy balance [26]. Ghrelin and leptin control appetite and energy balance [27], while SCFAs produced by gut microbiota activate GPR41 and GPR43 receptors on enteroendocrine cells (EECs), influencing GLP-1 secretion, which regulates glucose metabolism and suppresses appetite [28, 29]. Adiponectin and resistin, hormones produced by adipose tissue, are linked to lipid metabolism and insulin resistance, contributing to obesity [30]. LPS is a key factor in inducing inflammation, exacerbating metabolic disturbances and promoting obesity [3, 31]. Modifying any of these compounds could potentially help regulate homeostatic processes and alleviate obesity-related disorders. McFarlin et al. suggest that Bacillus-based probiotics can lower postprandial ghrelin levels, improving appetite control, and reduce endotoxin levels, including LPS, potentially offering anti-inflammatory effects [27]. Similarly, the meta-analysis by da Silva Borges indicates a reduction in ghrelin levels with inulin-type fructan supplementation [32]. Gut microbiota, through metabolites like SCFAs, can influence GPR41 and GPR43 activation, regulating metabolism and reducing inflammation, which is important in the context of obesity [30]. Microbiota modification may also affect the expression of adiponectin and resistin genes, impacting thermogenesis and fat oxidation [33]. Although the literature suggests that probiotic therapy could modulate this key compounds related to obesity, our results did not confirm these effects. Moreover, there are studies consistent with our findings that showed no impact of probiotic therapy on LPS [34] or adipokines [35].

As discussed in our previous publication, the effect of probiotic therapy may be overshadowed by the substantial impact of prehabilitation and surgery on the microbiome and clinical outcomes [9]. In the bariatric treatment pathway, there are many opportunities for drastic changes in the microbiome, such as perioperative preparation, the procedure itself, and restrictive caloric reduction [36, 37]. Moreover, we assessed the effect of probiotic therapy as a preoperative preparation on postoperative outcomes, during a period when probiotic therapy had already been discontinued. Therefore, the effects of probiotic therapy on the microbiome might not have occurred or not persisted until the follow-up. However, our findings suggest potential gut microbiota modulation and maintenance of effects six months post-surgery. We lack direct evidence and a comprehensive analysis of gut microbiota composition and activity, both of which would be highly beneficial. It is possible that the impact of probiotic therapy was too small to influence clinical outcomes but sufficient enough to affect cytokine secretion. By assessing the gut microbiota, we could potentially link specific effects to certain microorganisms and, consequently, aim to target bacterial strains to achieve desired outcomes.

Our study has several limitations that need to be considered. One of the primary limitations was the low number of patients included in the final statistical analysis, despite meeting the planned recruitment targets. Less than 50% of enrolled participants ultimately qualified for analysis, which reduced the study's power. The reasons for dropout included treatment discontinuation, meaning patients did not proceed to surgery (26.4% overall; 27.3% placebo, 25.5% probiotic), formal withdrawal from the study (5.5% overall; 3.6% placebo, 7.3% probiotic), non-adherence to the protocol, including unreported withdrawal or loss of contact (11.8% overall; 7.3% placebo, 16.4% probiotic), and loss to follow-up, where patients did not attend the control visit or failed to provide samples (17.3% overall; 18.2% placebo, 16.4% probiotics). Given that BS is not an urgent procedure, and probiotic supplementation is not a mandatory component of its therapy, a significant portion of patients either withdrew from the study or discontinued their treatment pathway. Patient drop-out is an inherent challenge when working with human subjects. While the average dropout rate in randomized trials is often reported to be around 30% [38], this challenge is particularly pronounced in long-term studies, such as ours, requiring active patient engagement and not involving immediate medical intervention. Retrospectively, a key logistical oversight identified was the distribution of study preparations to patients immediately upon recruitment. Considering financial and time constraints, a more effective strategy would have been to provide the preparations only after receiving initial baseline biological samples and data from the participants. This approach would have ensured that only engaged patients received the preparations, optimizing resource allocation and significantly increasing the likelihood of study completion.

We did not perform a detailed analysis of body composition changes, thus we cannot determine whether the changes in cytokines are related to changes in body composition. Additionally, there is a lack of comprehensive analysis of the gut microbiota composition and activity. Given the critical role that gut microbiota plays in regulating metabolic processes and inflammatory responses, the absence of this data limits our understanding of the mechanisms through which probiotic therapy may exert its effects. Moreover, biomarker assessment at the day of surgery was not performed, limiting our ability isolate the effects of preoperative probiotic supplementation from the metabolic and inflammatory effects of bariatric surgery. Furthermore, due to the low return rate of dietary diaries, we did not analyze the patients' dietary intake. However, the study was designed to minimize the impact of dietary variability on the results — the study was successfully randomized, and a standardized diet, tailored to the patients and supervised by a dietitian, was implemented. Another limitation is that the statistical power was only sufficient for detecting differences in IL-6 levels between the groups. This indicates that the study may not have been sensitive enough to detect significant differences in other parameters, potentially leading to Type II errors. Further studies with larger sample sizes may be necessary to fully understand the effects of probiotic therapy on these molecular markers.

However, by adjusting our significance level and calculating the power accordingly, we ensured that our findings are robust and minimize the risk of both Type I and Type II errors. This approach allows us to report the significant finding for IL-6 while acknowledging the limitations and speculative nature of the results for the other parameters. The study utilized a randomized controlled trial design, minimizing selection bias and enhancing the likelihood that the results are attributed to the intervention rather than to other factors. Moreover, the study analyzed a range of biomarkers providing a broad overview of the physiological changes associated with the probiotic therapy. To our knowledge, this is the first study to assess the effect of probiotic supplementation administered exclusively before bariatric surgery. The focus on the preoperative period allowed us to evaluate the potential of early microbiota modulation without the confounding effects of postoperative factors such as altered nutrient absorption, dietary restrictions, or antibiotic use. Our findings may serve as a reference point for future studies comparing different timing strategies for probiotic use in this population. Although the limited sample size and statistical power constrains the generalizability of present results, observed trends provide important insights for future studies. Our findings suggest that preoperative probiotic supplementation may modulate postoperative inflammation. These results underscore the need for further randomized controlled trials evaluating the optimal timing, duration, and composition of probiotic interventions in bariatric patients to determine their clinical value.

Probiotics, by modulating gut microbiota composition and activity, have the potential to influence various mechanisms involved in the regulation of body weight and metabolic health. Investigating the impact of probiotic interventions on tested compounds can shed light on the molecular mechanisms regulating appetite, gut health, and the pathogenesis of obesity. Reduction in IL-6 levels following preoperative probiotic therapy is an important finding that may have implications for future research and clinical practice. However, given the exploratory nature of this study and the limited statistical power, this study should be interpreted with caution as preliminary and hypothesis-generating. Further research is required to fully understand the complex relationships between gut microbiota, metabolic regulation, and weight management.

## Conclusions

In conclusion, preoperative probiotic therapy was associated with significantly lower IL-6 levels compared to placebo six months after surgery, suggesting a potential anti-inflammatory effect. However, since the between-group difference in IL-6 changes from baseline was not statistically significant, the observed effect should be interpreted with caution. Other inflammatory markers were not significantly affected. Nonetheless, given the low statistical power for these parameters, the possibility of undetected effects cannot be excluded. The potential effect of probiotics on the analysed markers appears to have been offset by the bariatric procedure itself, which significantly affected many of them. Further randomized controlled trials with a larger number of participants, addressing the limitations of our study, will be necessary to more precisely explore the effects of probiotics on molecular mechanisms regulating weight management in patients undergoing bariatric treatment.

## Data Availability

The data sets used and/or analyzed during the current study are available from the corresponding author upon reasonable request as we are currently working on additional publications from this project, and some of the data will be used in these forthcoming studies.

## Abbreviations

ALT: Alanine aminotransferase
ALP: Alkaline phosphatase
AST: Aspartate aminotransferase
BMI: Body mass index
BS: Bariatric surgery
CFU: Colony forming units
CRP: C-reactive protein
DM1: Type 1 diabetes mellitus
DM2: Type 2 diabetes mellitus
DL: Dyslipidemia
FFA: Free fatty acids
GAPDH: Glyceraldehyde-3-phosphate dehydrogenase
GGT: Gamma-glutamyl transferase
GLP-1: Glucagon-like peptide-1
HbA1c: Glycated hemoglobin
HDL: High-density lipoprotein
HOMA-IR: Homeostatic model assessment of insulin resistance
HT: Hypothyroidism
HTN: Hypertension
IL-2R: Interleukin-2 receptor
IL-6: Interleukin-6
LDH: Lactate dehydrogenase
LDL: Low-density lipoprotein
LPS: Lipopolysaccharide
LSG: Laparoscopic sleeve gastrectomy
OAGB: One anastomosis gastric bypass
OSAS: Obstructive sleep apnea syndrome
PCR: Polymerase chain reaction
qRT-PCR: Quantitative real-time polymerase chain reaction
SCFAs: Short-chain fatty acids
SOCS-3: Suppressor of cytokine signaling 3
TFF2: Trefoil family factor 2
TLR: Toll-like receptor
UCC: University Clinical Center
